# The implementation of Public–Private Partnership in China: A sustainable pathway?

**DOI:** 10.1371/journal.pone.0305051

**Published:** 2024-07-03

**Authors:** Chuan Zuo, Jun Li, Yatong Wang

**Affiliations:** 1 School of Finance and Public Administration, Shanghai Lixin University of Accounting and Finance, Shanghai, China; 2 College of Finance and Statistics, Hunan University, Changsha, Hunan, China; Budapest Metropolitan University of Applied Sciences, HUNGARY

## Abstract

The organizational forms of infrastructure in China are divided into two categories, the traditional Public Procurement Model (PUB) model and Public–Private Partnership(PPP) model. The main difference is the separation or binding of the construction and operation phases. A systematic understanding is needed of how Chinese local governments choose between these two models. In this paper, we take public capital congestion and local government objectives as the entry point to study the effects of both on PPP choice. Firstly, by constructing an endogenous economic growth model under the PPP model, and comparing it with the model under the PUB model, this paper initially explains how the rise in public capital congestion affects the choice of the PPP by growth-oriented local governments. Then the data from prefecture-level cities from 2009–2018 are utilized to conduct empirical tests. We find that urban economic growth pressures have a positive effect on the choice of PPP when the congestion of public capital increases. Furthermore, the implementation of PPP is indeed conducive to economic performance, and its core mechanism is to provide more infrastructure (like roads) rather than tax competition. The PPP model is more sustainable. We are the first to employ both modeling approach and the empirical research to address the implementation of Public–Private Partnership in China. And we have systematically analyzed the conditions and results of PPP selection by local governments. It formulates the Chinese PPP theory.

## Introduction

Public-Private Partnership (PPP) is originally launched by the British government in 1992 and operated as a Private Finance Initiative (PFI) to attract private capital for the building of new public infrastructure. The concept of PPP is much broader that can cover all types of collaboration across the private-public sector. PPP has not only been widely used in various transportation projects, but has also increasingly appeared in water, energy, communications, medical services, and education projects. At the beginning of the 21st century, China’s governments have also begun to actively promote the market-oriented reform of the public sector. They encouraged and supported non-public capital to participate in the investment, construction, and operation of various public utilities and infrastructure [1]. By the end of 2022, a total of 15,163 PPP projects had been transacted, with an investment amount of 20.92 trillion yuan and a completion rate of 76.93%. Supported by relevant policies, China has developed into the largest PPP market in the world.

The existing literature mainly discusses the determining factors that explain the use of PPP from the following dimensions. From the private sector, the first aspect is economic and financial [2]. PPP projects are characterized by non-recourse or limited recourse. The bank raises the interest rate on loans or provides only a small amount of loans to PPP projects. Financial and economic viability should be analyzed and assessed when the private sector decides on a project [3]. Additionally, when public funding is weaker, the credibility of the government’s ability to make long-term commitments decreases, and the uncertainty faced by the private sector increases, which can lead to the reluctance of the private sector to participate in PPP projects. It is necessary to increase public funding using new financing instruments [4] and innovative financing instruments [5].

The second is risk assessment. Private sector participation in PPP projects is subject to various risks. The government tends to transfer these risks to the private sector, resulting in the private sector being reluctant to participate in PPP projects. So an appropriate allocation of risks is a necessary condition for private sector participation in PPP projects [6]. Iossa and Martimortproposed that unbundling is better than bundling considering information asymmetries and moral hazard [7]. From the perspective of ownership distribution, the ownership of public goods should belong to the party that values their benefits the most in the incomplete contracts.

From the public sector, the first incentive is budgetary constraints. Governments often weigh the shadow cost of public funds against the loss of social welfare when deciding whether to adopt a PPP model or a traditional Public Procurement Methods (PUB) model. The stronger the budget constraint, the more distortionary the government will be in taxing taxpayers, and the higher the shadow cost of public funds will be [8]. Although information asymmetry between the government and the private sector implies a higher shadow cost of public funds, BOT (Build-Operate-Transfer) programs can reduce the shadow cost of public funds. Empirical studies also show that governments are more inclined to adopt the PPP model when they face budget constraints and financial difficulties [9, 10].

The second one is political incentives. Given that decision-making on public service delivery rests with the government (politicians), PPP rely on the motivations of political leaders and public administrators. Officials also consider PPP as a tool to obtain political rent. The degree of rent is determined by the degree of political competition [11]. But essentially, it just serves partisan objectives. PPP tends to be used by left-wing governments using the data of 80 low- and middle-income countries over the period. Ross and Yan noticed the drive behind the behavior and attempt to explain this issue based on the government’s objectives, such as maximizing value for money or maximizing total social surplus [12].

The Chinese central government’s hierarchical political achievement incentive system establishes promotional competitions among local authorities, using the gross domestic product (GDP) as the key standard [13]. Under this mode, local governments are under pressure to grow their economies in order to win promotion competitions. Infrastructure determines the level of regional economic development, and PPP is the main form of organization for building infrastructure. However, few studies have focused on the impacts of the pressure created by economic growth targets on the choice of PPP. This paper attends to explain how economic growth target pressures can play a key role in China’s PPP development and sustainability of this development model.

Firstly, this paper establishes an analysis framework for the impact of economic growth target pressures on the choice of PPP. Whether separating building and operating phases is the main characteristic between PPP and PUB. PPP is associated with bundling the various tasks, whereas PUB assigns each task to a single independent private partner which the government outsources [14, 15]. Based on these characteristics, this paper constructs an endogenous economic growth model of PPP to analyze the government’s behavior in maximizing economic growth targets. Compared with the models of PUB, this paper concludes that the choice of PPP is influenced by the interaction effect of economic growth pressure and the congestion of public capital. PPP is more sustainable than PUB in promoting economic growth.

In the empirical analysis, we manually gathered the annual government work reports of China’s provincial governments and municipal governments from 2009 to 2018 and cited the annual economic growth goal values. The target pressure increase the likelihood of choosing PPP when the degree of congestion rises. To deal with the endogeneity problem, we exploit Instrumental Variables (IV) and use a Two-Stage Least Squares (2SLS) strategy. The results still hold. Furthermore, we find that the implementation of PPP is indeed conducive to economic performance, and its core mechanism is to provide more infrastructure (such as roads) rather than engage in tax competition. This development model is more sustainable. The empirical results are consistent with our theoretical hypothesis and framework.

The contributions of this paper to the literature are as follows. Regarding the research perspective, this paper analyzes the impact of economic growth pressure on the choice of PPP from a macro-perspective based on the key characteristics between PPP and PUB. So far, there has been no systematic analysis about it. In addition, regarding research methods, this paper empirically assesses the impact of economic growth pressure on the choice of PPP using a modeling approach, and the empirical research involves discussing endogeneity problems. This paper constructs IV for economic growth pressure, thereby avoiding estimation bias caused by endogeneity issues. The modeling approach provides a more intuitive representation of the mechanism. Additionally, this paper integrates Chinese characteristics into empirical analysis, such as official tenures, PPP acceleration terms, PPP categories, and the location of PPP. It formulates the Chinese PPP theory.

The rest of this paper is arranged as follows. Section 2 presents the institutional background for setting economic growth targets. Section 3 lays out the theoretical analysis framework. Section 4 outlines econometric models, data, and variables. Section 5 reports the results of the empirical analysis. Finally, Section 6 presents the conclusion.

## Institutional background

According to China’s fiscal system (*Budget Law of the People’s Republic of China*), the allocation of central and local expenditure responsibilities is based on administrative affiliation. But theoretically, the allocation should be based on the scope of public service spillovers, resulting in fiscal responsibilities that should theoretically be borne by the central government being borne by local governments. Along with the reform of the fiscal system (*Decision of the State Council on the Implementation of the Tax-Sharing Fiscal Management System*), local fiscal revenues have declined, and fiscal burdens have increased. To fulfill fiscal responsibility, local governments need to raise fiscal revenue and the excess fiscal revenue can be retained. From this point of view, the local government’s goal should be to maximize fiscal revenue rather than maximize economic growth. However, maximizing economic growth can be facilitated by maximizing fiscal revenue. Specifically, the fiscal revenue of local governments consists of two parts: budgetary revenue and extra-budgetary revenue. Tax is the main component of budgetary revenue and land revenue is the main part of extra-budgetary revenue. First, local economic growth will undoubtedly lead to an increase in tax revenues. Second, economic growth will eventually be internalized in the appreciation of land value.

The decentralization of economic management authority is one of the important factors that prompted the transformation of the target. Since 1982 (*Resolution on the institutional reform of the State Council*), the central government has begun to decentralize part of economic management authority to localities. This series of initiatives has made it possible for local governments to maximize fiscal revenues by promoting economic growth. However, at the same time, this reform also makes local governments enjoy a certain degree of discretionary power. Local governments can use their power to encroach on the interests of residents and enterprises to obtain revenue. They may not need to make any effort to develop the economy. Coupled with the fact that information asymmetry limits the vertical accountability of local officials, and inadequate horizontal supervision makes it difficult for residents with information superiority to play a supervisory role. It is entirely possible for discretionary power to evolve into the right to do legitimate harm. Under such circumstances, the core of government governance lies in how to design a set of incentive mechanisms to transform the hand that seizes into help. The personnel appraisal system (*Regulations on the Examination Work of Party and Government Leaders and Cadres*), which focuses on economic growth performance, completes the design of this set of incentives and further establishes the goal of local governments as maximizing economic growth.

## Theoretical analysis and resea rch hypothesis

Earlier studies are more likely to consider public capital as a pure public good. They focus on the transfer paths and optimal fiscal policies in dynamic equilibrium states when public capital enters the production function in different forms. Barro and Sala-I-Martin suggested that almost all public capitals have different degrees of congestion [16]. The congestion of public capital must be considered in the endogenous economic growth model, otherwise, the results will be a large deviation.

Dioikitopoulos and Kalyvitis considered public capital congestion and drew an endogenous economic growth model to describe PUB [17]. In this model, the government sector financs both public investment and maintenance. Meanwhile, maintenance expenditure affected the depreciation of public capital. This section provides an endogenous economic growth model based on Dioikitopoulos and Kalyvitis [17] to analyze PPP. The model’s main features are as follows: (a) the government sector finances public investment while the private sector maintains it. (b) because of the combination of building and operation, the technology of maintenance is higher than PUB. So decay of public capital decelerates. Compared to these two models, this section attemps to explain what determines the choice of PPP.

### Model

#### The representative agent

The economy consists of N homogeneous consumer-producers. There is no population growth and unitizes N to 1. The representative consumer consumes c to maximize the following utility function (1):

$$\begin{array}{l} max\int_{0}^{\infty }u(c)e^{-\rho t}dt\\ u(c)=ln\;c \end{array}$$ (1)

The representative producer’s production function (2) is as follows:

$$\begin{array}{l} y=k^{\alpha }{\left(K_{g}^{s}\right)}^{1-\alpha }\\ K_{g}^{s}=K_{g}{\left(\frac{k}{K}\right)}^{\sigma } \end{array}$$ (2)

The production good y is determined by private capital k and public capital K^s^_g_. Public capital is congested due to the use of private capital, and σ represents the degree of congestion. When σ = 0, public capital is like pure-public goods, which are non-rival and non-excludable. Whereas σ>0, private capital will increase the proportion of aggregate private capital to obtain more public capital. But the maximum vaule of σ is 1.

It has been argued that public capital congestion mainly arises from the use of private capital. Like roads and railroads, the public services that private capital receives from these infrastructures are directly related to individual use. In a decentralized economy, an individual’s perception is that his actions are sufficiently small that he will not affect the economy as a whole. It treats the total amount of capital as given when making decisions about its investment behavior. When there is congestion in productive public capital, individuals believe that by increasing their private investment, they can compete for more public capital to serve their production. Then, when the impulse to invest exists for all individuals, it creates overinvestment and further aggravates the congestion of public capital for the economy as a whole.

The level of private capital accumulation is determined by investment i. To simplify the analysis, we ignore the depreciation of private capital. The constraint on private capital accumulation is then given by the following Eq (3):

$$\dot{k}=i$$ (3)

### Public capital

The level of public capital accumulation is determined by investment I_g_ and depreciation σ_g_, is presented by Eq (4)_._ The government invests in public capital while the private sector maintains it to slow down its depreciation. The government is financed by tax revenue and the government budget constraint, is given by Eq (5). The after-tax output of the representative agent is used to invest in private capital, consume and maintain public capital, is presented by Eq (6):

$$\begin{array}{c} {\dot{K}}_{g}=I_{g}-\delta_{g}(\frac{m_{g}}{y})K_{g}\\ \delta {'}_{g}()<0;\delta '{'}_{g}()<0;\delta_{g}()\in ({\bar{\delta }}_{g},1)\\ \frac{m_{g}}{y}=\mu_{g}(1-\tau )\\ \delta_{g}={\left[\mu_{g}(1-\tau )\right]}^{-\xi } \end{array}$$ (4)

$$\tau Y=I_{g}$$ (5)

$$(1-\tau )y=i+c+m_{g}$$ (6)

Where m_g_ denotes the maintenance expenditure of the representative agent. The depreciation function σ_g_() is negatively influenced by maintenance expenditure and output ratio. Where ξ denotes the elasticity of μ_g_ to σ_g_, which indicates the level of maintenance technology. Conversely, σ_g_() is positively influenced by output y due to the use of public capital. τ denotes the tax rate.

### The representative agent problem

The current-value Hamiltonian of the representative agent is given by Eq (7)

$$H=u(c)+\lambda [(1‐\tau )y‐c‐m_{g}]+\gamma [I_{g}-\sigma_{g}(\frac{m_{g}}{y})K_{g}]$$ (7)

Where λ denotes the marginal value of private capital for the representative agent. Accordingly, γ denotes the marginal value of public capital to the representative agent. We assume λ is equal to γ. The first-order conditions are given by:

u'(c)=λ (8)

$$-1=\delta {'}_{g}\frac{K_{g}}{y}$$ (9)

$$(1-\tau )\frac{\partial y}{\partial k}=-\delta {'}_{g}\mu_{g}\frac{K_{g}}{y}\frac{\partial y}{\partial k}+\rho -\frac{\dot{\lambda }}{\lambda }$$ (10)

$$(1-\tau )\frac{\partial y}{\partial K_{g}}=-\delta {'}_{g}\mu_{g}\frac{K_{g}}{y}\frac{\partial y}{\partial K_{g}}+\delta_{g}+\rho -\frac{\dot{\gamma }}{\gamma }$$ (11)

Where:

$$\frac{\partial y}{\partial k}=(\alpha +\sigma (1-\alpha ))k^{\left(1-\alpha )(\sigma -1\right)}K^{-\sigma (1-\alpha )}K_{g}^{1-\alpha }$$ (12)

$$\frac{\partial y}{\partial K_{g}}=(1-\alpha )k^{\alpha +\sigma (1-\alpha )}K^{-\sigma (1-\alpha )}K_{g}^{-\alpha }$$ (13)

The Eq (8) shows that the marginal utility of consumption equals the shadow price of private capital. The Eq (9) represents the conditions for optimal maintenance expenditure. The Eq (10) states that the optimal after-tax marginal product of private capital is equal to its opportunity cost. The opportunity cost of private capital includes the marginal effect of investment in private capital on public capital depreciation, time preference, and the gain of private capital. The marginal effect of investment in private capital on public capital depreciation is positive, which is caused by the fact that an increase in private capital investment reduces maintenance expenditures. Similarly, Eq (11) reflects that the optimal after-tax marginal benefit of public capital is equal to its opportunity cost, which includes the marginal effect of an increase in public capital on public capital depreciation, public capital depreciation, time preference, and the gain of public capital. The marginal effect of an increase in public capital on public capital depreciation is positive. This is because of the congestion of public capital, private capital will increase the proportion of aggregate private capital to obtain more public capital and reduce maintenance expenditure on public capital. The marginal product of private capital and public capital are presented by Equs (12)–(13).

According to the first-order conditions, we can obtain Eq (14):

$$\begin{array}{l} (1-\mu_{g})(1-\alpha -az)(1‐\mu_{g})=-\frac{\delta_{g}}{\delta_{g}^{'}}\\ z=\frac{K_{g}}{K},a=\alpha +\sigma (1-\alpha ) \end{array}$$ (14)

The optimal maintenance proportion is given by Eq (15):

$${\hat{\mu }}_{g}=\frac{1-\alpha -az}{\frac{1}{\xi }+1-\alpha -az}$$ (15)

The level of maintenance technology has a positive impact on the optimization of maintenance ratio ξ. The higher the maintenance technology level, the greater the marginal benefits. As a result, the private sector tends to increase maintenance spending. Conversely, the level of congestion σ hampers optimal maintenance ratios. The more congestion there is, the more willing the private sector is to invest to obtain more public capital and reduce maintenance spending. Similarly, the higher the ratio of public capital to private capital, the private sector prefers to increase investment rather than maintenance.

### Balanced growth and equilibrium dynamics

The Balanced Growth Path (BGP) describes a state where all economic variables grow constantly. Based on the basic assumption and model, the growth rate of economic variables is given by functions (16)-(18).


$$\frac{\dot{C}}{C}=(1-\tau )(1-{\overset{⌢}{\mu }}_{g})az^{1-\alpha }-\rho$$ (16)

$$\begin{array}{c} \frac{\dot{K}}{K}=(1-\tau )(1-{\overset{⌢}{\mu }}_{g})z^{1-\alpha }-w\\ w=\frac{C}{K} \end{array}$$ (17)

$$\frac{{\dot{K}}_{g}}{K_{g}}=\tau z^{-\alpha }-\delta_{g}$$ (18)

The necessary condition for BGP, where all economic variables grow at the same rate is satisfied, by analyzing the equilibrium growth rates of consumption, private and public capital. If the consumption growth rate is constant, then private and public capital need to grow at the same rate, denoted as g_k_ = g_Kg_ = g. Given the equation, for the growth rate of consumption to be constant, it is still need that both private capital and consumption grow at the same rate, say g_c_ = g_k._ We can obtain that g_c_ = g_k_ = g_Kg_ = g, which also satisfies the equation.

Next, we examine the existence and stability of this dynamic system:

$$\frac{\dot{w}}{w}=\frac{\dot{C}}{C}-\frac{\dot{K}}{K}=-(1-\tau )(1-\mu_{g})(1-\alpha )(1-\sigma )z^{1-\alpha }-\rho +w$$ (19)

$$\frac{\dot{z}}{z}=\frac{{\dot{K}}_{g}}{K_{g}}-\frac{\dot{K}}{K}=-(1-\tau )(1-\mu_{g})z^{1-\alpha }+\tau z^{-\alpha }-\delta_{g}+w$$ (20)

BGP requires that these two Eqs (19)–(20) be equal to zero, we can obtain Eq (21):

$$\begin{array}{c} \Theta (z^{*})=(1-\tau )(1-\mu_{g})(\alpha +\sigma (1-\alpha ))z^{{*}^{1-\alpha }}-\tau z^{{*}^{-\alpha }}-\rho +\delta_{g}\\ \Theta '(z^{*})=(1-\tau )(1-\mu_{g})(\alpha +\sigma (1-\alpha ))z^{{*}^{-\alpha }}+\alpha \tau z^{{*}^{-\alpha -1}}\\ -(1-\tau )\frac{\partial \mu_{g}}{\partial z}[(\alpha +\sigma (1-\alpha ))z^{{*}^{1-\alpha }}-\delta_{g}^{'}]>0\\ \Theta (0)=\delta_{g}-\rho ;\underset{z\to \infty }{lim}\Theta =+\infty \end{array}$$ (21)

According to the Intermediate Value Theorem, this dynamic economic system has a unique equilibrium (z*, w*).

In the end, we perform a first-order linear expansion of the dynamical system equations at equilibrium (z*, w*). The determinant of this system is given by Eq (22):

$$\begin{array}{l} |J|=-a(1-\tau )(1-\mu_{g})(1-\alpha )w^{*}z^{{*}^{1-\alpha }}\\ +(1-\tau )w^{*}z^{{*}^{2-\alpha }}(1-(1-\alpha )(1-\sigma ))\\ -\tau \alpha w^{*}z^{{*}^{-\alpha }}-{\delta^{'}}_{g}(1-\tau )w^{*}z^{*}(\frac{\partial \mu_{g}}{\partial z})<0 \end{array}$$ (22)

The Eq (22) indicates that the dynamic system is local saddle point stable.

### Growth-maximizing policies

In this section, we analyze the fiscal policies when the government aims at maximizing growth. The government chooses tax rate τ to maximize the economy’s growth rate. Meanwhile, the government knows how a private’s reaction to the fiscal policy.

The maximum problem is shown by Eq (23):

$$max(1-\tau )(1-{\hat{\mu }}_{g})az^{1-\alpha }-\rho$$ (23)

Taking the first-order conditions and using the Eq (15), we can obtain the optical conditions which are presented by Eqs (24)–(26):

$$\frac{1}{\hat{\tau }}=1+\frac{\alpha }{\left(1-\alpha )(1-{\hat{\mu }}_{g}\right)}$$ (24)

$$-{\delta^{'}}_{g}={\hat{z}}^{-\alpha }$$ (25)

$$(1-{\hat{\mu }}_{g})(1-\alpha -az)=\frac{1}{\xi }{\hat{\mu }}_{g}$$ (26)

Where parameters ρ, σ, and ξ determine the optimal solution of μ_g,_ τ, z. Then we take the total differential of the equation to analyze the effect of parameter σ on the endogenous variables, which is presented by Eq (27):

$$\begin{array}{l} \left(\begin{array}{ccc} v_{11} & v_{12} & 0\\ v_{21} & v_{22} & v_{23}\\ v_{31} & 0 & v_{33} \end{array}\right)\left(\begin{array}{l} d{\hat{\mu }}_{g}\\ d\hat{\tau }\\ d\hat{z} \end{array}\right)=\left(\begin{array}{l} 0\\ 0\\ (1-{\hat{\mu }}_{g})(1-\alpha )\hat{z} \end{array}\right)d\sigma \\ v_{11}=(1-\alpha )(1-\hat{\tau });v_{12}=\alpha +(1-{\hat{\mu }}_{g})(1-\alpha );\\ v_{21}=-{\delta^{''}}_{g}(1-\hat{\tau });v_{22}={\delta^{''}}_{g}{\hat{\mu }}_{g};v_{23}=\alpha {\hat{z}}^{-\alpha -1};\\ v_{31}=-(1-\alpha -a\hat{z})-\frac{1}{\xi };v_{33}=-(1-{\hat{\mu }}_{g})a \end{array}$$ (27)

The determinant of the coefficient matrix is presented by Eq (28):

$$\begin{array}{l} |J|=-{\delta^{''}}_{g}(1-\hat{\tau })(1-{\hat{\mu }}_{g})a\\ -(1-\alpha -a\hat{z}+\frac{1}{\xi })(\alpha +(1-{\hat{\mu }}_{g})(1-\alpha ))\alpha {\hat{z}}^{-\alpha -1}<0 \end{array}$$ (28)

The effect of parameter σ on the optimal solution of μ_g,_ τ, z is presented by Eqs (29)–(31):

$$\frac{d\hat{\tau }}{d\sigma }=-\frac{1}{\left|J\right|}\alpha {\hat{z}}^{-\alpha }(1-\hat{\tau }){\left(1-\alpha \right)}^{2}(1-{\hat{\mu }}_{g})>0$$ (29)

$$\frac{d{\hat{\mu }}_{g}}{d\sigma }=\frac{1}{\left|J\right|}\alpha {\hat{z}}^{-\alpha }(1-\alpha )(1-{\hat{\mu }}_{g})[(1-\alpha )(1-{\hat{\mu }}_{g})+\alpha ]<0$$ (30)

$$\frac{d\hat{z}}{d\sigma }=\frac{1}{\left|J\right|}{\delta^{''}}_{g}(1-\alpha )(1-\hat{\tau })\hat{z}(1-{\hat{\mu }}_{g})<0$$ (31)

### What determines the choice of government?

How the government chooses between PUB and PPP is influenced by the degree of congestion and pressure on economic growth targets.

When the government chooses PPP, if the level of congestion increases, the private sector will increase the investment to acquire public capital and reduce maintenance expenditure. So the depreciation of public capital will accelerate. However, the depreciation of public capital will reduce the ratio of public capital to private capital, and the private sector will increase maintenance expenditure. In the end, the optimal maintenance ratio will decrease according to the equation. In this situation, the government will increase the tax rate and invest in public capital to maximize the economy’s growth rate. The economy’s growth relies on private and public capital.

When the government chooses PUB, the government will reduce maintenance expenditure if the degree of congestion grows. This is because the construction phases are separated from the operation phases, the maintenance technology is relatively low, and the marginal benefit of maintenance is also relatively low. Consequently, the tax rate will decrease [17]. In this situation, the economy’s growth only relies on private investment. The government won’t have the incentive to increase the tax rate to encourage private investment. However, as congestion increases, this growth pattern is unsustainable, and the government will choose PPP.

When the level of public capital congestion is high, the higher the pressure on the economic growth of local governments, the more they tend to choose PPP projects. When the degree of public capital congestion is low, the greater the pressure on local government economic growth, the more uncertainty exists regarding whether to choose PPP projects. Because both PPP and PUB models can achieve the goal of maximizing economic growth.

The conclusion based on the model is summarized in the following hypothesis:

**Hypothesis:** Whether the government chooses PPP is influenced by economic growth pressures and public capital congestion, with a positive moderating effect of congestion.

## Materials and methods

### Dataset

For the empirical analysis, we assembled a city-level dataset that contains comprehensive indicators collected from four major sources.

First, we collect the government work reports at the prefectural and provincial levels that announce economic growth targets approved by the local people’s congresses. The main source of government working reports is the government web portal. Furthermore, the reports are also published in the “China City Statistical Yearbook” as a special issue.

Our second data source is CPPPC (China Public Private Partnerships Center) which contains all PPP projects that require expenditures from the government in China. We use information from this dataset on PPP’s number, investment, industry, and stage at the city level.

Thirdly, we obtain the features of a municipal party secretary (such as the age and tenure of officials) from the Official Government Website, Baidu Encyclopedia, and People’s Daily Online. If more than one municipal party secretary serves in a city in a given year, only the longest-serving one is retained.

The fourth major data source is the “China City Statistical Yearbook” and Wind, which contain rich information on cities’ basic characteristics. We extract GDP per capita, density, fiscal revenue, fiscal expenditure, loans, value added of the secondary sector, value added of the third sector, investment in fixed assets, area, and government debt.

Particularly, the congestion of public capital is caused by the use of private capital. So the congestion of public capital is ideally measured by the ratio of private capital to public capital. However, the “China City Statistical Yearbook” contains only data on the overall investment in fixed assets, not divided into private investment or public investment. But data at the provincial level identify the source of investment in fixed assets, allowing us to recognize fixed-asset investment by state-holding and collective-holding as public investment. Based on that, we use the perpetual inventory method with 2000 as the base period to measure the public capital stock. The results are shown in Table 1. Since 2009, most of the public capital percentage has been below 50%, thus the level of total capital stock aggregation can reflect the level of congestion to a certain extent. Therefore, at the prefecture level, we also use 2000 as the base period, calculating the capital stock level by the perpetual inventory method, and then calculating the capital agglomeration by LQ (location quotient).

**Table 1:** Public capital percentage at prefectural level.

Public capital percentage	2009	2010	2011	2012	2013	2014	2015	2016	2017
**Beijing**	41%	38%	36%	33%	32%	31%	30%	27%	26%
**Tianjin**	41%	42%	42%	40%	39%	36%	35%	31%	28%
**Hebei**	41%	38%	35%	32%	29%	27%	25%	23%	21%
**Shanxi**	51%	51%	49%	47%	46%	44%	43%	39%	37%
**Inner Mongolia**	42%	41%	39%	38%	37%	37%	38%	39%	39%
**Liaoning**	33%	31%	29%	27%	25%	24%	23%	22%	21%
**Jilin**	33%	32%	30%	28%	27%	26%	26%	24%	23%
**Heilongjiang**	48%	46%	44%	42%	39%	37%	35%	33%	31%
**Shanghai**	46%	45%	43%	41%	39%	36%	35%	31%	29%
**Jiangsu**	36%	32%	30%	28%	26%	26%	25%	23%	21%
**Zhejiang**	37%	35%	33%	31%	30%	29%	29%	27%	25%
**Anhui**	39%	36%	33%	31%	29%	28%	26%	25%	24%
**Fujian**	39%	38%	36%	35%	34%	32%	32%	30%	28%
**Jiangxi**	39%	36%	33%	30%	28%	27%	25%	24%	22%
**Shandong**	37%	34%	32%	29%	27%	26%	24%	22%	21%
**Henan**	35%	32%	29%	26%	24%	22%	21%	19%	18%
**Hubei**	51%	47%	43%	39%	36%	33%	31%	29%	28%
**Hunan**	43%	41%	38%	36%	35%	33%	33%	31%	29%
**Guangdong**	35%	35%	33%	31%	29%	28%	26%	24%	23%
**Guangxi**	38%	37%	34%	32%	30%	29%	28%	27%	26%
**Hainan**	35%	33%	31%	30%	28%	26%	26%	23%	22%
**Chongqing**	38%	38%	38%	37%	36%	35%	34%	31%	28%
**Sichuan**	44%	43%	41%	39%	38%	37%	36%	35%	34%
**Guizhou**	54%	51%	49%	46%	45%	45%	46%	41%	39%
**Yunnan**	57%	54%	50%	47%	45%	44%	45%	46%	47%
**Shannxi**	56%	54%	52%	50%	48%	47%	46%	45%	45%
**Gansu**	62%	60%	58%	56%	54%	52%	50%	49%	48%
**Qinghai**	54%	52%	51%	50%	49%	48%	51%	50%	50%
**Ningxia**	43%	40%	39%	36%	34%	33%	33%	32%	31%

Table 2 provides summary statistics, including the outcome variables: the number of PPP projects; regressor of interest: economic growth targets; moderator variable: capital congestion; and a set of city-level characteristics, which are used for controls.

**Table 2:** Summary statistics.

Variable	Obs	Mean	Std. Dev.	min	max
**outcome variables:**					
the number of PPP project (piece)	2065	3.275	5.784	0.000	74.000
**regressor of interest:**					
economic growth pressures(%)	2065	10.008	2.575	1.000	25.000
**moderator variable:**					
capital congestion	2065	1.997	5.199	0.025	222.496
**cities’ characteristics:**					
governments debt / GDP	2065	155.038	329.888	-109.000	3446.180
GDP per capital	2065	56405.350	144276.786	8477.000	6421762.000
density(person/km2)	2065	467.970	322.050	9.787	2648.110
fiscal revenue/expenditure	2065	0.501	0.227	0.069	1.541
loan/GDP	2065	1.143	0.969	0.165	16.743
value added of third sector/ value added of secondary sector	2065	0.922	0.463	0.109	5.340
official’s age(year)	2065	68.624	172.212	42.000	2018.000
official’s tenure(year)	2065	4.193	1.776	1.000	10.000
square of the official’s tenure	2065	20.731	17.133	1.000	100

### Empirical specification

Based on the theoretical analysis in section 3, we construct an empirical model to derive testable hypotheses. To identify the effect of economic growth targets on the choice of PPP, the baseline estimation model is shown by Eq (32):

$$y_{it}=\alpha +\beta_{1}g_{it}+\beta_{2}X_{it}+\delta_{i}+\lambda_{t}+\varepsilon_{it}$$ (32)

Where i, t donate city and year, respectively.*y*_*it*_ is the number of PPP projects. *g*_*it*_ is economic growth pressures of local governments, *X*_*it*_ is a set of controls that includes information about the cities’ characters. *δ*_*i*_ is the city-fixed effect that captures all city-specific and time-invariant characteristics. λ_*t*_ is year fixed effects, controlling for all nationwide shocks common to cities in a particular year. *ε*_*it*_ is the error term.

Based on the benchmark regression, we further introduce an interaction term (*c*_*it*_) between capital congestion and economic growth pressures to verify the moderating effect. The estimation model is shown by Eq (33):

$$y_{it}=\alpha +\beta_{1}g_{it}+\beta_{2}c_{it}+\beta_{3}X_{it}+\delta_{i}+\lambda_{t}+\varepsilon_{it}$$ (33)

Poisson Regression and Negative Binomial Regression are mainly used to deal with cases where the explanatory variables are count variables. However, Poisson regression requires the data to meet meet the condition of equal dispersion (mean and standard deviation are equal). If the data is likely to produce over-dispersion phenomenon, that is, if the data’s mean and standard deviation are obviously not equal, the use of negative binomial regression is better. In our studies, *y*_*it*_ is the number of PPP projects, which is a count variables. According to Table 2, the average number of PPP projects is 3.275, and the standard deviation is 5.784. The mean is smaller than the standard deviation. Negative Binomial Regression should be used instead of Poisson Regression. But Poisson Regression is used in robust checks.

Besides, Poisson Regression and Negative Binomial Regression can’t solve the endogenous issue, such as two-way causality. Two-way causality means that the choice of PPP may increase the economic growth pressures of local governments. Because PPP projects take a relatively long time to generate economic benefits. Therefore, we construct the IV for economic growth pressure and use IV estimation to address the endogenous issue.

First, we choose the number of cities in the province. Because the greater the number of cities, the more intense the competition subject to the constraint of limited promotional positions, and thus the greater the competitive pressure on economic growth targets. Futhermore, the number of prefectures remains constant during the sample period. It is determined by the central government and is relatively exogenous. Meanwhile, to control for time effects, we construct an interaction as an IV between the number of cities in the province and the average value of the province’s economic growth target over the next two years. Second, we select the weighted average of economic growth targets of other cities in the same province. Local governments tend to react strategically to other ‘competitors’ behavior to stand out in promotion tournaments. So that the economic growth targets of other sister cities within the same province increase the pressure on economic growth targets, but do not directly impact other economic variables.

The estimation strategy consists of two steps. First, we use two IV and their interaction term with economic congestion to estimate using Eq (34):

$$\begin{array}{l} g_{it}=\alpha_{1}+\beta_{11}z_{it1}+\beta_{12}z_{it2}+\beta_{13}z_{it1}w_{it}+\beta_{14}z_{it2}w_{it}+\gamma_{1}X_{it}+\delta_{1i}+\lambda_{1t}+\varepsilon_{1it}\\ c_{it}={\alpha^{'}}_{1}+{\beta^{'}}_{11}z_{it1}+{\beta^{'}}_{12}z_{it2}+{\beta^{'}}_{13}z_{it1}w_{it}+{\beta^{'}}_{14}z_{it2}w_{it}+{\gamma^{'}}_{1}X_{it}+{\delta^{'}}_{1i}+{\lambda^{'}}_{1t}+{\varepsilon^{'}}_{1it} \end{array}$$ (34)

Where z_it1_ represents the interaction term between the number of cities in the province and the average value of the province’s economic growth target over the next two years. z_it2_ is the weighted average of economic growth targets of other cities in the same province. w_it_ is the capital congestion. **X**_it_ is the control variable that accounts for the characteristics of city i in year t. δ_1i_ reflects the city effect, and λ_1t_ represents the time effect.

The next step is the key estimation Eq (35):

$$y_{it}=\alpha_{2}+\beta_{21}{\hat{g}}_{it}+\beta_{22}{\hat{c}}_{it}+\gamma_{2}X_{it}+\delta_{2i}+\lambda_{2t}+\varepsilon_{2it}$$ (35)

Where y_it_ is the economic outcome of the city i in year t. ${\hat{g}}_{it}$ and ${\hat{c}}_{it}$ are the predicted variables from the first-stage estimations. Similarly, X_it_ is the control variable that reflects the characteristics of the city i in year t. δ_2i_ reflects the city effect and λ_2t_ represents the time effect.

In addition, we conduct a battery of robustness checks for identification assumptions in Section 5.2, using an alternative measurement of PPP, excluding the impact of non-landed projects, etc.

## Empirical results

### Baseline estimate

Table 3 reports the basic regression results, where column (1) shows the regression results with only economic growth pressures, city, and year-fixed effects. Column (2) further controls for municipal party secretary characteristics and the prefectural city’s economic characteristics based on column (1). The regression results show that economic growth pressures do not affect the choice of the PPP, as reflected by the effect of the economic growth target on the number of PPP is not significant. According to the analysis of the theory in part 3, local governments can maximize their economic growth objectives by adjusting their fiscal policies in both PPP and PUB models. The effect of the economic growth target on the choice of the PPP is influenced by other factors.

**Table 3:** Baseline estimate.

	(1)	(2)	(3)	(4)
Variable	the number of PPP project	the number of PPP project	the number of PPP project	the number of PPP project
**economic growth pressures**	0.046	0.039	0.044	0.021
	(1.44)	(1.22)	(1.39)	(0.66)
**economic growth pressures**			0.001**	0.012***
* capital congestion			(2.50)	(2.86)
**governments debt / GDP**		0.000***		0.000***
		(2.89)		(3.66)
**GDP per capita**		0.000**		-0.000***
		(2.20)		(-2.69)
**density**		0.000		-0.000
		(0.04)		(-1.27)
**fiscal revenue/expenditure**		0.032		0.025
		(0.09)		(0.07)
**loan/GDP**		0.102***		0.048
		(3.25)		(1.33)
**value added of third sector/ value added of secondary sector**		-0.190**		-0.165*
		(-2.01)		(-1.77)
**official’s age**		-0.000		-0.000
		(-0.98)		(-0.84)
**official’s tenure**		-0.014		-0.010

To verify the moderating effect of public capital congestion, the interaction terms of capital congestion and economic growth target are introduced. Column (3) only contains regression results of the economic growth target, the interaction terms, and city and year fixed effects. While column (4) further controls for the municipal party secretary’s characteristics and the city’s economic characteristics based on column (3). The regression results indicate that the selection of PPP projects by economic growth targets is positively moderated by capital congestion.

Under the PUB model, local governments have no incentive to increase tax revenue to maintain infrastructure. Instead, they adopt a tax competition strategy, i.e., lowering the tax burden to attract more capital in order to maximize economic growth. While under the PPP model, the marginal return on maintenance rises due to the unification of the construction and maintenance phases. Maintenance of public capital is the responsibility of the private sector, which, to a certain extent, prevents the reduction of the level of depreciation of public capital by private capital. At the same time, local governments maximize their economic growth targets by increasing tax revenues and investing in infrastructure. Compared to the PUB model, the PPP model is more sustainable in achieving economic growth objectives when the level of capital congestion rises. Therefore, the effect of economic growth pressures on the choice of PPP project model is moderated by the degree of infrastructure congestion. Hypothesis is valid.

Congestion of public capital has increased as the demand for public capital has risen. The objectives of local governments, determined by the political and economic system, have prompted them to choose the PPP model. This shows that the Chinese institutional environment is more favorable for the development of PPP. It also theoretically and empirically demonstrates the intrinsic and extrinsic motivations for the rapid development of PPP in China.

### Robust check

#### Endogenous issue

To minimize the interference of endogeneity in the estimation results, the IV method is further used to regress the benchmark model. Table 4 illustrates the estimation results of the IV approach. Columns (1) and (2) display the regression results of the first stage. In column (1), the regression coefficients of the two IV (iv1, iv2) are significantly positive at a 1% level of significance. From column (2), the regression coefficients of the cross terms of the two IV are significantly positive at a 1% level of significance. Column (3) shows the results of the second-stage regression, and the results of the IV approach are in agreement with the results of the baseline empirical evidence, which again validates hypothesis.

**Table 4:** IV estimate.

	(1)	(2)	(3)
Variable	Stage1	Stage1	Stage2
	economic growth pressures	economic growth pressures	the number of PPP project
		* capital congestion	
**iv1**	0.011***	0.007	
	(2.76)	(0.78)	
**iv2**	0.752***	-0.176**	
	(20.58)	(-2.23)	
iv1* capital congestion	0.001*	0.008***	
	(1.82)	(5.13)	
iv2* capital congestion	-0.031***	0.788***	
	(-3.14)	(36.76)	
**economic growth pressures**			0.003
			(0.04)
**economic growth pressures**			0.027*
* capital congestion			(1.86)
**Control**	YES	YES	YES
**Year_Fe**	YES	YES	YES
**Fixed_Fe**	YES	YES	YES
**N**	1525.00	1525.00	1488.00

### Alternative measurements of PPP

The previous analysis measures the dependent variable by the number of PPP projects, while the investment scale of PPP projects itself can also reflect the favor of local governments. Therefore we also consider the proportion of PPP investment to GDP as an explanatory variable. The specific regression results are shown in columns (1) and (2) of Table 5.The relevant conclusions still exist, and the regression results confirm the robustness of our research conclusions.

**Table 5:** Alternative measurements of PPP.

	(1)	(2)
Variable	invest2	invest2
**economic growth pressures**	0.002	0.002
	(0.93)	(0.76)
**economic growth pressures**	0.000***	0.001***
* capital congestion	(3.69)	(4.86)
**Control**	NO	YES
**Year_Fe**	YES	YES
**Fixed_Fe**	YES	YES
**N**	2065.00	2065.00

### Alternative ways to estimate

First, we replace the Negative Binomial Regression with Poisson Regression, and the specific estimation results are shown in columns (1) and (2) of Table 6. Second, whether PPP projects introduced in a prefecture-level city in a certain year is also a very important aspect of portraying the choice of PPP. We assign the value of 1 to a prefecture-level city if there are PPP projects in a certain year; otherwise, the value of 0 is assigned. The results of the above regression are consistent with the conclusion of the benchmark regression. The regression analysis in this paper is very robust to the choice of different methods.

**Table 6:** Poisson and Probit estimate.

	(1)	(2)	(3)	(4)
Variable	Poisson	Poisson	Probit	Probit
**economic growth pressures**	0.035	0.024	0.135*	0.110
	(1.49)	(0.99)	(1.93)	(1.51)
**economic growth pressures**	0.001***	0.007**	0.017*	0.037**
* capital congestion	(3.21)	(2.36)	(1.90)	(2.54)
**Control**	NO	YES	NO	YES
**Year_Fe**	YES	YES	YES	YES
**Fixed_Fe**	YES	YES	YES	YES
**N**	2065.00	2065.00	1555.00	1555.00

### Control the impact of non-landed projects

Since many PPP projects in the Treasury’s Management Pool do not land that is in the identification, preparation, and procurement stages. That may lead to an overestimation of our sample size and could impact our findings. In this section, we only retain the projects in the implementation stage or handover stage, and the results of the regression are as shown in columns (1) and (2) of Table 7. The results of the robustness tests still support the conclusion that economic growth pressures impact the choices between PPP and PUB, positively moderated by public capital congestion.

**Table 7:** Exclude projects that have not landed.

	(1)	(2)
Variable	number	number
**economic growth pressures**	0.037	0.000
	(0.98)	(0.01)
**economic growth presssures**	0.001**	0.017***
* capital congestion	(2.22)	(3.64)
**Control**	NO	YES
**Year_Fe**	YES	YES
**Fixed_Fe**	YES	YES
**N**	1812.00	1812.00

### Heterogeneity analysis

#### Officials tenures

The average tenure of a municipal party secretary is approximately 4 years, and we group regressions using the 4-year cutoff. The results are as shown in Table 8. Columns (1) and (2) of Table 8 present the regression results with an average tenure of fewer than 4 years, and columns (3) and (4) of Table 8 present the results with an average tenure greater than or equal to 4 years. The regression results show that the moderating effect of public capital congestion will not be significant when the officials’ tenure exceeds the average tenure. This is because promotion prospects are expected to be stronger at the beginning of an official’s tenure than at the end of the tenure. So when the tenure is shorter than the average tenure, officials are more motivated to develop the economy, and the interaction effect between economic growth pressures and public capital congestion is more significant. Conversely, when approaching the end of the tenure, promotion prospects are weak, and the interaction between them is correspondingly insignificant.

**Table 8:** Heterogeneous responses by officials’ tenures.

	(1)	(2)	(3)	(4)
Variable	the number of PPP project	the number of PPP project	the number of PPP project	the number of PPP project
**economic growth pressures**	-0.034	-0.072	0.085*	0.063
	(-0.64)	(-1.33)	(1.86)	(1.36)
economic growth pressures *capital congestion	0.001	0.014*	0.001**	0.006
	(0.17)	(1.86)	(1.99)	(0.93)
**Control**	NO	YES	NO	YES
**Year_Fe**	YES	YES	YES	YES
**Fixed_Fe**	YES	YES	YES	YES
**N**	665.00	665.00	1178.00	1178.00

#### PPP acceleration terms

The development of China’s PPP accelerated significantly after 2013. We analyze the heterogeneous effects by dividing the years into two subgroups, based on whether the year is after 2013. The regression results are shown in the Table 9. Columns (1) and (2) of Table 9 show the regression results before 2013, and columns (3) and (4) of Table 9 show the regression results after 2013. The regression results indicate that the interaction effect of economic growth pressure and public capital congestion is more significant after 2013.

**Table 9:** Heterogeneous responses by PPP acceleration(after 2013).

	(1)	(2)	(3)	(4)
Variable	the number of PPP project	the number of PPP project	the number of PPP project	the number of PPP project
**economic growth pressures**	-0.034	-0.072	0.066*	0.041
	(-0.64)	(-1.33)	(1.95)	(1.19)
**economic growth pressures**	0.001	0.014*	0.001**	0.011***
* capital congestion	(0.17)	(1.86)	(2.41)	(2.63)
**Control**	NO	YES	NO	YES
**Year_Fe**	YES	YES	YES	YES
**Fixed_Fe**	YES	YES	YES	YES
**N**	665.00	665.00	1230.00	1230.00

#### Industry category

The PPP projects contain 19 industry categories, some of which, such as social security, are not directly linked to economic performance. To test the hypotheses, we further differentiate the PPP projects into economic and social projects. The regression results are shown in Table 10. Columns (1) and (2) of Table 10 show the regression results for social-type projects, while columns (3) and (4) of Table 10 show the regression results for economic-type projects. The regression results show that the interaction effect plays a more significant role in the selection of PPP projects under the economic category.

**Table 10:** Heterogeneous responses by PPP industry category.

	(1)	(2)	(3)	(4)
Variable	the number of PPP project	the number of PPP project	the number of PPP project	the number of PPP project
**economic growth pressures**	0.045	-0.074	0.093***	0.069*
	(0.41)	(-0.60)	(2.59)	(1.88)
**economic growth pressures**	-0.012	0.000	0.001**	0.015***
* capital congestion	(-0.77)	(0.01)	(2.35)	(3.20)
**Control**	NO	YES	NO	YES
**Year_Fe**	YES	YES	YES	YES
**Fixed_Fe**	YES	YES	YES	YES
**N**	681.00	681.00	1875.00	1875.00

#### Target accomplishment

If officials do not achieve the economic growth target in the previous year, they will have greater incentives to promote economic growth in the following year, achieve the set economic growth target, and be more inclined to select PPP projects. To test the hypothesis, this paper categorizes prefecture-level cities according to whether they achieved their economic growth targets in the previous year. Columns (1) and (2) of Table 11 show the regression results for accomplishing the economic growth target, and columns (3) and (4) of Table 11 show the regression results for not accomplishing the economic growth target. The regression results show that the interaction effect of the economic growth target and public capital congestion is more significant in the selection of PPP projects when officials have not accomplished the previous year’s economic growth target.

**Table 11:** Heterogeneous responses by target adjustment.

	(1)	(2)	(3)	(4)
Variable	the number of PPP project	the number of PPP project	the number of PPP project	the number of PPP project
**economic growth pressures**	0.041	0.038	0.013	-0.011
	(0.56)	(0.49)	(0.31)	(-0.25)
**economic growth pressures**	0.013**	0.017	0.002**	0.013**
* capital congestion	(2.07)	(1.59)	(2.37)	(2.23)
**Control**	NO	YES	NO	YES
**Year_Fe**	YES	YES	YES	YES
**Fixed_Fe**	YES	YES	YES	YES
**N**	930.00	930.00	1060.00	1060.00

### The location of the PPP

Due to the wide disparities in regional economic development in China, the location of the PPP can also influence the results. This article divides the sample into eastern, middle, and western regions based on the cities where the PPPs are located. Column (1) of Table 12 shows the regression results for the eastern region. Results are consistent with the hypothesis. Column (2) of Table 12 shows the regression results for the middle region. The moderating effect of public capital congestion is not significant; only the effect of economic growth pressures on PPP selection is significant. This means that governments in the middle region choose PPP under economic growth pressures driven by other factors, such as financing needs. Column (3) of Table 12 shows the regression results for the western region. The moderating effect of public capital congestion and the effect of economic growth pressures are not significant. Possibly due to the underdeveloped regional economy and weak private capital, the government prefers the PUB model.

**Table 12:** Heterogeneous responses by the location of PPP.

	(1)	(2)	(3)
Variable	the number of PPP project	the number of PPP project	the number of PPP project
**economic growth pressures**	-0.056	0.150**	0.042
	(-1.18)	(2.17)	(0.65)
**economic growth pressures**	0.016**	0.000	0.005
* capital congestion	(2.55)	(0.07)	(0.51)
**Control**	YES	YES	YES
**Year_Fe**	YES	YES	YES
**Fixed_Fe**	YES	YES	YES
**N**	881.00	646.00	531.00

## Further analysis

Based on the previous analysis, we conclude that local governments, under the pressure of economic growth targets, are more inclined to choose PPP projects when the degree of public capital congestion rises to obtain better economic performance. Furthermore, we want to know whether officials can bring better economic performance after choosing PPP projects. We test it using the event study method. The estimation Eq (36) is as follows [18]:

$$y_{it}=\alpha +\beta_{1}\sum D_{\_xi}+\ldots \beta_{i}D_{xi}+\delta_{i}+\lambda_{t}+\varepsilon_{it}$$ (36)

Where y_it_ is the economic variable that we are concerned about. First, y_it_ is the economic outcome of the city i in year t, which is measured by the nighttime lighting data. This data is considered a good indicator to measure the level of economic outcomes. Second, if PPP is conducive to economic outcomes, we want to know whether it is through tax competition or increasing infrastructure. And then y_it_ is the tax burden of enterprise and the highway mileage in the city i of year t. D__xi_ is a dummy variable that equals one, meaning all observations in the city i that are x years before the first chosen PPP project. While D_xi_ equals one for the year after first choosing in city i. Similarly, X_it_ is the control variable that reflects the characteristics of city i in year t. δ_i_ reflects the city effect that does not vary over time. λ_t_ represents the time effect that does not change with the individual. The result is shown in Table 13.

**Table 13:** The effect of PPP.

	(1)	(2)	(3)
Variable	lnlight	mean burden	lnroadlong
**d_4**	-0.026	0.001	-0.004
	(-0.88)	(0.07)	(-0.25)
**d_3**	-0.081***	0.002	-0.023
	(-2.84)	(0.26)	(-1.62)
**d_2**	-0.062**	-0.003	-0.017
	(-2.07)	(-0.30)	(-1.14)
**d_1**	0.000	0.000	0.000
	(.)	(.)	(.)
**d1**	0.083**	0.008	0.031*
	(2.29)	(0.81)	(1.66)
**d2**	0.148***	0.000	0.049**
	(3.29)	(0.03)	(2.15)
**d3**	0.185***	-0.005	0.112***
	(3.40)	(-0.32)	(4.05)
**d4**	0.223***	-0.005	0.158***
	(3.37)	(-0.26)	(4.71)
**d5**	0.273***	0.040	0.141***
	(3.18)	(1.61)	(3.22)
**d6**	0.248**	0.029	0.066
	(2.18)	(0.91)	(1.15)
**_cons**	8.907***	0.087***	5.963***
	(96.97)	(2.89)	(126.50)
**Control**	YES	YES	YES
**Year_Fe**	YES	YES	YES
**Fixed_Fe**	YES	YES	YES
**N**	2733.00	1784.00	2694.00

From column (1), it can be concluded that using the year of PPP implementation as the base period, the economic performance of the economy is lower than that of the current period before the PPP implementation and higher than that of the current period after the implementation, with a certain degree of sustainability. From columns (2) and (3), the implementation of PPP mainly promotes local economic performance by affecting infrastructure development rather than the tax burden of regional firms.

## Discussion

To investigate the critical factors of Public–Private Partnership choice in China, this paper summarizes the objectives of the Chinese local governments, establishes an endogenous economic growth model under PPP, and analyzes circumstances under which an economic growth-oriented government would choose PPP model rather than PUB model. The key hypotheses are derived from the theoretical analysis: Whether government chooses PPP is influenced by the economic growth pressures and public capital congestion, there is a positive moderating effect of congestion. The empirical tests also verify it.

Additionally, we believe that the burden on economic growth goals intensifies when officials start their term and the prior year’s targets remain unachieved. Under intense pressure on economic growth goals, the impact of public capital congestion on selecting PPP becomes more significant. This confirms the hypothesis that the selection of PPP is shaped by the pressures of economic expansion and congestion in public capital. Furthermore, the influence of PPP traits on our hypotheses, including the duration of development, classification, and geographical setting, is taken into account. Our findings confirm the validity of the hypothesis when PPP is measured in terms of acceleration, within the economic classification, and situated in the eastern region.

Our research verifiy that PPP projects in China are government-led. The conclusion confirms Chan et al.’s finding that the most critical risks of PPP projects in China are related to the government [19]. The government can not only reduce the risk of the private sector by providing government guarantees [20], but also can increase the risk of the private sector through government intervention, corruption and poor public decision-making processes [19]. It is important to implement effective measures that can reduce project risks and attract more high-quality private capital. At the same time, it should also avoid providing excessive guarantees that could over-benefit the private sector and lead to project failure. Thereby capitalizing on the advantages of government-led PPP.

Second, this study finds that PPP to be more often used by governments that aim to maximize economic growth, especially when public capital congestion increases in China. This result is more specific compared to the viewpoint that PPP is a public governance scheme aimed at maximizing public interest [21]. Additionally, the research analyzes the choice of PPP based on the main characteristics between PPP and PUB. PPP is associated with bundling various tasks, whereas PUB assigns each task to a single independent private partner that the government outsources to. The analytical perspectives are contrary to the research from a micro-level perspective, such as the concession period of PPP [22].

Third, we found evidence that the government, driven by economic growth pressure and public capital congestion, prefers to choose PPP in economic infrastructure rather than social infrastructure. This also provides evidence for why risk studies mainly focus on economic infrastructure [23].

## Conclusion

This paper takes another look at the causes of PPP choice from a new perspective of growth target pressure. Firstly, we analyze the political-economic system with Chinese characteristics and how it affects the local government’s objectives. Then we establish a an endogenous economic growth model under PPP and compared it with the model under PUB. To analyzed the relationship between economic growth pressures and the choice of PPP. Besides we conduct the conditions under which economic growth pressures have an effect on the choice of PPP. Later, we collect relevant datas and examined the hypothesis.

We find that urban economic growth pressures didn’t have significantly promoted the choice of PPP. However, it has a positive effect when the congestion of public capital increases. Comparatively, the effect is more substantial in cities that during the early term of officials, with economic PPP projects and have not accomplished the target last year. In addition, the effect is more significant after 2013 when the PPP accelerated. Furthermore, we observed that the implementation of PPP is beneficial to economic performance. The core mechanism is to increase the infrastructure, such as the road, rather than decrease the tax burden of firms in the cities. This model is more sustainable.

This paper has several policy implications. First, local governments have their own logic for choosing the form of infrastructure organization and have fully considered the local constraints. Therefore, it is not appropriate for the central government to engage in one-size-fits-all control of local PPP projects, but to make full use of the information advantages and positive initiatives of local governments. Second, PPP projects in China are local government-led, the success of PPP projects in China is related to the local government. The central government should play a regulatory role, such as improving the legislative and supervisory systems for PPP, transparency in decision-making, and so on. Third, particularly in the central and western regions, PPP should be prevented from becoming a tool for local government financing and increasing the risk of local government debt. Controlling the government’s annual expenditure responsibly within a certain range, in line with the local economic development, past and future government expenditure status. So as to measure its own fiscal space and use this as a basis for setting the budget threshold for investment in PPP projects. In addition, local government objectives depend largely on the central government’s assessment and incentive mechanism for local governments, so it is more appropriate for the central government to intervene by improving the central government’s assessment and incentive mechanism for local governments. Then indirectly optimize the local government objectives and the local government’s choice of infrastructure organization. In particular, it is important to explore how to make PPPs work better in the social infrastructure sector.
